# Spermatic vein thrombosis with lupus anticoagulant, a cause of acute inguinal pain: a case report

**DOI:** 10.11604/pamj.2020.36.125.20448

**Published:** 2020-06-25

**Authors:** Mohammed Aynaou, Tarik Mhanna, Amine Elhoumaidi, Paapa Dua Boateng, Ali Barki

**Affiliations:** 1 Department of Urology, Mohamed VI University Hospital Center, Mohamed First Oujda, Morocco

**Keywords:** Spermatic vein, thrombosis, lupus anticoagulant

## Abstract

Patients with lupus anticoagulants are at high risk of systemic arterial and venous thrombosis and arterial stroke. We present an unusual case of a young man presenting inguinal pain. Doppler ultrasound revealed spermatic vein thrombosis on the left side. Hematologic workup revealed positive lupus anticoagulant. The patient was treated with therapeutic heparin.

## Introduction

Spermatic vein thrombosis is a rare event which can be difficult to diagnose. Multiple predisposing factors have been associated with Spontaneous Spermatic vein thrombosis, like malignant tumors, coagulopathies and varicocele [1]. We present a case of spermatic vein thrombosis in the left spermatic vein.

## Patient and observation

**Figure 1:**
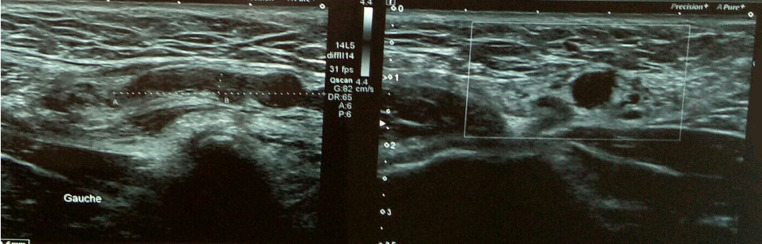
ultrasonography demonstrated tubular structure hypoechoic non compressed consistent with thrombus

**Figure 2:**
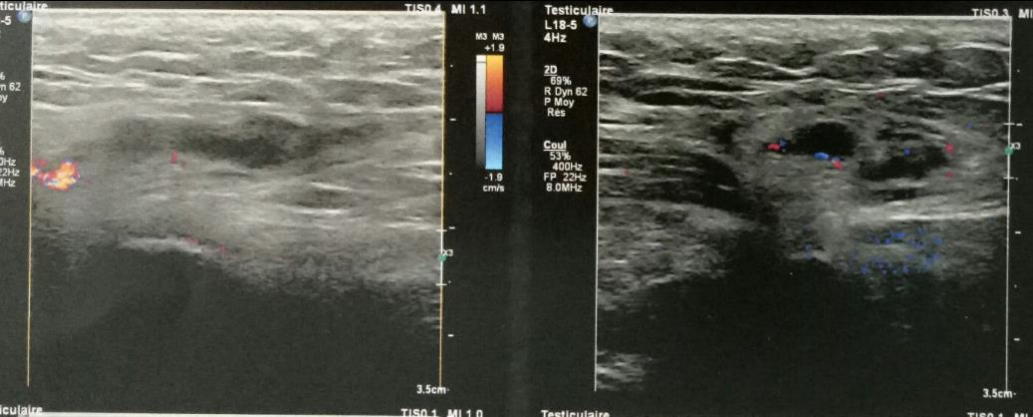
color Doppler signal was not presented inside the hypoechoic structure

**Figure 3:**
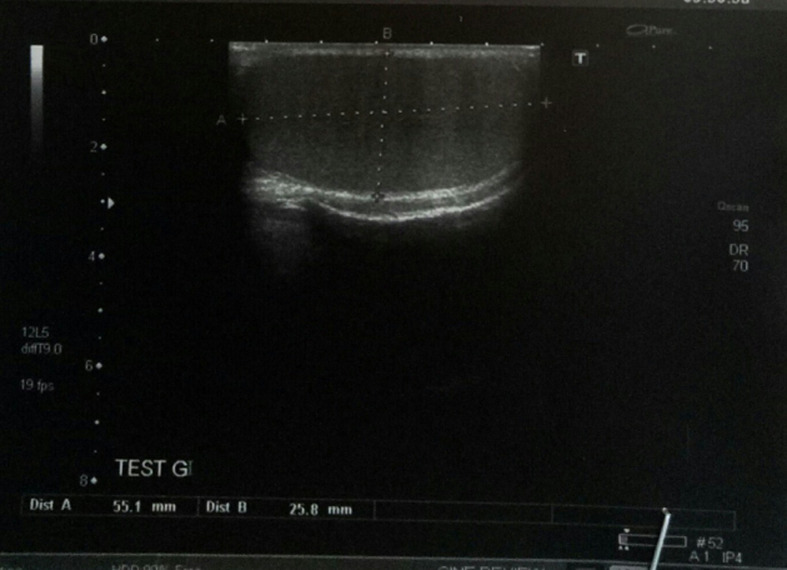
left testicular without abnormality

**Figure 4:**
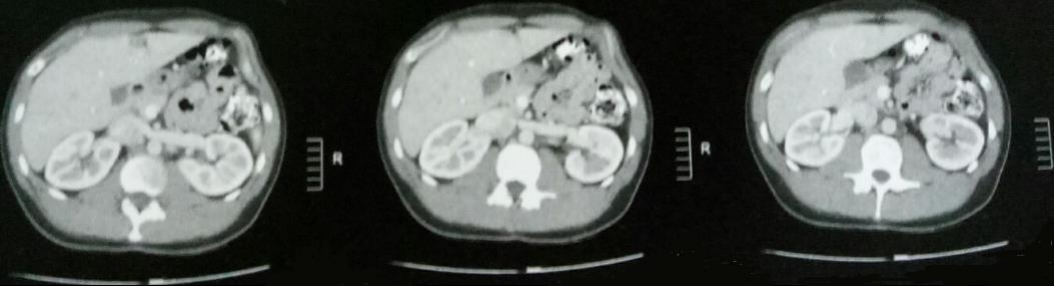
absence of renal masses on the abdominal CT scan

A 27-year-old male, with no previous health problems, was admitted with a 1-week history of painful in the left inguinal region, without fever or any associated signs. He denied any etiologic factors like trauma, surgeries, severe exercise or thrombogenic factors. Inguinal region, scrotum and prostate were normal to palpation. Initial laboratory test, were normal. Doppler ultrasonography of left inguinal region demonstrated an expanded vein which contained thrombus (Figure 1) with no blood flow at Doppler (Figure 2). Scrotal Doppler ultrasound (Figure 3) and Total abdomen contrast-enhanced CT examination excluded other diseases (Figure 4). Complete biology workup was negative, except lupus Anticoagulant was positive. A medical treatment of anticoagulant at a curative dose was started. After 15 days, the patient´s inguinal pain was completely disappeared. Three months later, inguinal ultrasound revealed no residual evidence of spermatic vein thrombosis.

## Discussion

Spermatic vein thrombosis is a rare pathology, it can clinically simulate an incarcerated hernia [2]. In addition, there is another differential diagnosis such as spermatic cord torsion, benign and malignant tumors of spermatic cord [3-5]. Several etiologic factors are associated with spermatic vein thrombosis, likes trauma to the vascular endothelium, obstruction to venous drainage, hypercoagulable states, vigorous sexual activity or sport activity, infection, tumors of the genitor urinary tract and inguinal hernia surgery etc. [6]. Doppler ultrasound offers a non-invasive and accurate means of establishing and confirming the diagnosis. Lupus anticoagulants are associated with an increased incidence of venous and arterial thrombotic events [7-9]. In the literature we report cases of venous thrombosis of pulmonary [9], retinal [10], renal [11] and cerebral [12]. Several mechanisms of thrombosis induced by lupus anticoagulant includes antiphospholipid activity [13], inhibition of prostacyclin formation [14], prekallikrein inhibition [15] and direct injury of the vessel wall by an antibody-antigen complex [8]. The management of thrombosis of spermatic vein is controversial. For Thrombosis venous localized out of external inguinal ring we can propose conservative management including watchful observation. Whereas for deep seated spermatic vein thrombus inside the external inguinal ring, surgical approach May prevent pulmonary embolism. Anticoagulant therapy can be used clinically.

## Conclusion

Spermatic vein thrombosis is particularly rare disease. Ultrasound should be the first line examination to avoid exploratory surgery.
